# Funnel-hood-assisted endoscopic retrograde appendicitis therapy for acute appendicitis

**DOI:** 10.1055/a-2241-8907

**Published:** 2024-02-15

**Authors:** Qingtian Luo, Shaoxiong Zeng, Minwen Jiang, Qu Zhang, Chunsheng Cheng

**Affiliations:** 1 Department of Gastroenterology and Endoscopy Center, Huazhong University of Science and Technology Union Shenzhen Hospital (Nanshan Hospital), Shenzhen, China

Acute appendicitis is one of the most common acute abdominal conditions in the clinic, and is characterized by an acute onset, severe symptoms, and rapid progression 1 . Currently, endoscopic retrograde appendicitis therapy (ERAT) is an emerging treatment method for acute appendicitis, which involves colonoscopically guided intubation of the appendix, drainage of pus, imaging to clarify the diagnosis, flushing of the lumen, placement of drainage, and so on, to relieve the obstruction and reduce the pressure in the appendiceal lumen 2 . ERAT has the advantages of less trauma, shorter operation time, shorter hospital stay, lower complication rate, faster postoperative recovery, preservation of appendiceal immunity, and absence of surgical scar 2 3 . However, the conventional transparent hood and guidewire used for ERAT, the contrast catheter, and other instruments do not enter the appendiceal lumen easily, often leading to failure of the operation, and this limits the popularity and widespread adoption of ERAT 4 .

We report here the case of a 33-year-old man diagnosed with acute appendicitis and referred for ERAT. Colonoscopy showed marked hyperemia and swelling of the mucosa around the appendiceal orifice ( Fig. 1 ). We have simplified and modified the ERAT by using an independently developed funnel-shaped hood with a small-diameter tip ( Fig. 2 ) which facilitates insertion of the hood into the appendiceal lumen for the subsequent maneuvers ( Fig. 3 , Video 1 ). The patient’s abdominal symptoms completely disappeared after funnel-hood-assisted ERAT, and there was no pressure or rebound pain in the abdomen on physical examination. The patient was discharged on the 3rd postoperative day.

**Fig. 1:**
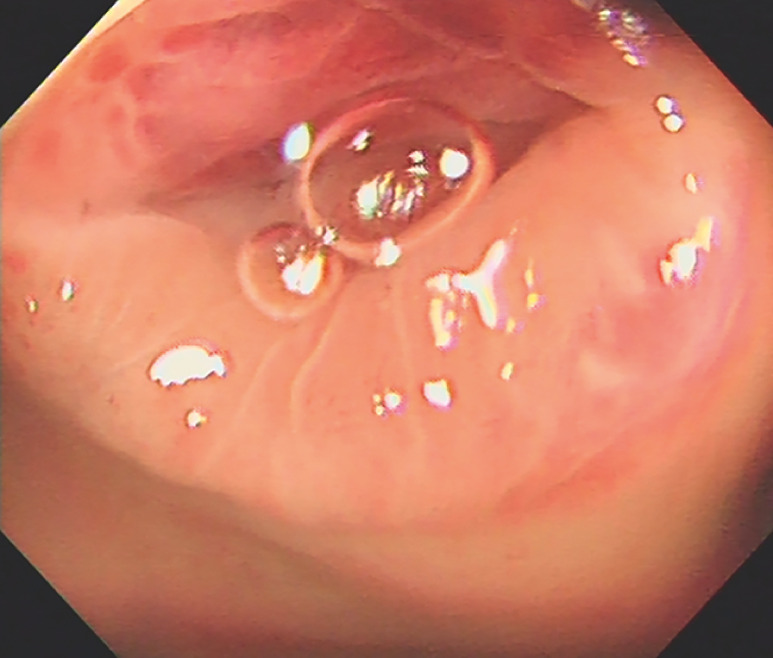
Colonoscopy in a 33-year-old man diagnosed with acute appendicitis showed marked hyperemia and swelling of the mucosa around the appendiceal orifice.

**Fig. 2:**
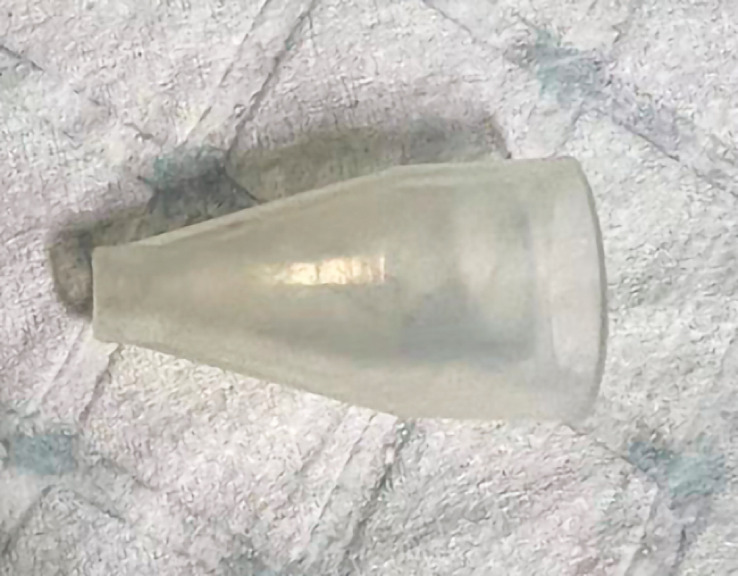
Photograph of the funnel-shaped hood.

**Fig. 3:**
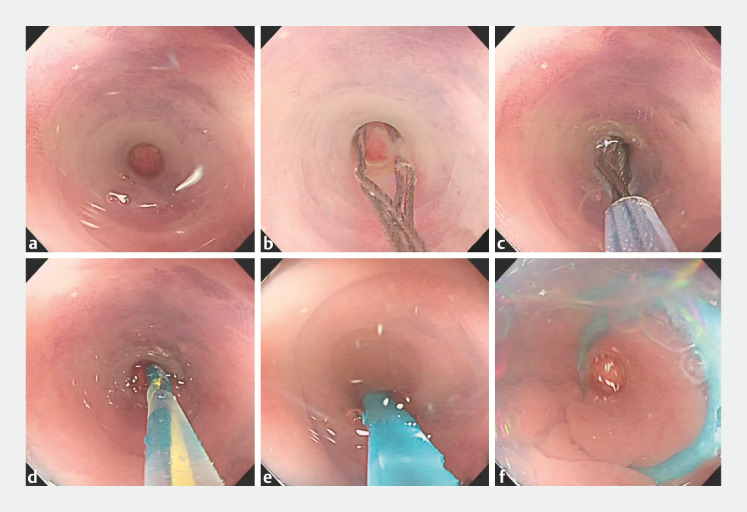
Funnel-hood-assisted endoscopic retrograde appendicitis therapy. **a** The Gerlach flap of the appendiceal opening was opened using a funnel-shaped hood, and the appendiceal orifice was exposed and secured. **b** The mesh basket was applied to align the appendiceal orifice in order to carry out blunt detachment and intubation; the mesh basket was inserted into the appendiceal lumen, and purulent secretion was seen to flow out. **c** The appendiceal lumen was repeatedly rinsed with saline until no pus or fecal material remained to come out and the rinsing fluid was seen to be clear. **d** The appendiceal orifice was intubated with a zebra guidewire. **e** Purulent secretion can be seen flowing from the appendix during placement of a drainage tube. **f** A well-placed drainage tube.

Funnel-hood-assisted endoscopic retrograde appendicitis therapy for acute appendicitis.Video 1

Funnel-hood-assisted ERAT makes appendiceal intubation less difficult and is a technological innovation that may be expected to improve the treatment success rate of ERAT and thus to help popularize this treatment.

Endoscopy_UCTN_Code_TTT_1AQ_2AF

## Notes

Endoscopy E-Videos https://eref.thieme.de/e-videos
*E-Videos* is an open access online section of the journal *Endoscopy* , reporting on interesting cases and new techniques in gastroenterological endoscopy. All papers include a high-quality video and are published with a Creative Commons CC-BY license. Endoscopy E-Videos qualify for HINARI discounts and waivers and eligibility is automatically checked during the submission process. We grant 100% waivers to articles whose corresponding authors are based in Group A countries and 50% waivers to those who are based in Group B countries as classified by Research4Life (see: https://www.research4life.org/access/eligibility/ ). This section has its own submission website at https://mc.manuscriptcentral.com/e-videos .

## References

[1] Moris D Paulson EK Pappas TN Diagnosis and management of acute appendicitis in adults: a review JAMA 2021 326 2299 2311 10.1001/jama.2021.20502 34905026

[2] Ding W Du Z Zhou X Endoscopic retrograde appendicitis therapy for management of acute appendicitis Surg Endosc 2022 36 2480 2487 10.1007/s00464-021-08533-8 33983458

[3] Yang B Kong L Ullah S Endoscopic retrograde appendicitis therapy versus laparoscopic appendectomy for uncomplicated acute appendicitis Endoscopy 2022 54 747 754 10.1055/a-1737-6381 35021234 PMC9329065

[4] Liu BR Ma X Feng J Endoscopic retrograde appendicitis therapy (ERAT): a multicenter retrospective study in China Surg Endosc 2015 29 905 909 10.1007/s00464-014-3750-0 25106722

